# A Successfully Treated Case of Gastrointestinal Stromal Tumor Causing Severe Anemia and Localized Peritonitis Showing Angina Pectoris Resulting in Watershed Cerebral Infarction

**DOI:** 10.1155/2017/6030561

**Published:** 2017-08-20

**Authors:** Yoshihide Sehara, Yuka Hayashi, Kenichi Ohya, Naoki Kaneko, Mikio Sawada

**Affiliations:** ^1^Department of Neurology, Ishibashi General Hospital, 628 Ishibashi, Shimotsuke, Tochigi 329-0596, Japan; ^2^Division of Genetic Therapeutics, Center for Molecular Medicine, Jichi Medical University, 3311-1 Yakushiji, Shimotsuke, Tochigi 329-0498, Japan; ^3^Department of Neurology, Jichi Medical University, 3311-1 Yakushiji, Shimotsuke, Tochigi 329-0498, Japan; ^4^Department of Cardiology, Shin-Oyama Shimin Hospital, 2251-1 Hitotonoya, Oyama, Tochigi 323-0827, Japan; ^5^Department of Neurosurgery, Jichi Medical University, 3311-1 Yakushiji, Shimotsuke, Tochigi 329-0498, Japan

## Abstract

Ischemic stroke following acute myocardial infarction is a rare but a serious complication. Because the pathophysiology of stroke is dynamic, it is often hard to identify the cause of stroke. Here, we present the case of a 75-year-old man with ischemic stroke following angina pectoris caused by severe anemia and localized peritonitis due to gastrointestinal stromal tumor of small intestine. On admission, he showed consciousness disturbance, fever, and left hemiplegia. The electrocardiogram on admission showed ST-segment depression in V2 to V6 which was normalized 4 hours later. The ultrasound cardiogram showed the mild hypokinesis in the apical portion of left ventricle which was also normalized later. The magnetic resonance imaging and angiography showed ischemic stroke in watershed area between right anterior and middle cerebral arteries area and stenosis of distal portion of right middle cerebral artery. The computed tomography of abdomen showed a mass of small intestine. We decided to perform curative surgery after transfusion and successfully resected the mass of the small intestine, which was revealed to be a gastrointestinal stromal tumor (GIST). This is a successfully treated case of GIST in which the complicated pathophysiology of watershed cerebral infarction following angina pectoris might be clearly revealed.

## 1. Introduction

Though it is not common, ischemic stroke followed by an acute myocardial infarction (AMI) is well known to be a serious complication. In general, several cardiac disorders, such as atrial fibrillation, mitral valve disease, and AMI, are thought to be the frequent cause of ischemic stroke [1]. However, the underlying mechanism of ischemic stroke in conjunction with heart disease is often hard to identify.

Gastrointestinal stromal tumors (GISTs) are defined as stromal tumors of the gastrointestinal tract, with a spindle cell, epithelioid, or occasionally pleomorphic morphology, which are potentially malignant [2]. The predominant localization for GISTs is the stomach (60%) and small intestine (20–30%). The incidence is estimated to be approximately 1–1.5 per 100,000 per year [3]. Many GISTs grow asymptomatically until they are identified clinically because of abdominal pain (35–40%), gastrointestinal bleeding (15–25%) and subsequent anemia (15–30%), mass pressure effects, or perforation [4, 5].

In this case, we present a man with a GIST and potential right middle cerebral artery stenosis, who suffered a watershed cerebral infarction following angina pectoris during sleep. We hypothesize that severe anemia caused by the bleeding from the intestine and the localized peritonitis caused by the GIST triggered an insufficient oxygen supply to myocardium. Because the man showed no cardiac symptoms after transfusion and more than 4 metabolic equivalents (METs) on a treadmill test, he underwent radical resection of the GIST. This appears to be a successfully treated case of GIST in which the complicated pathophysiology of watershed cerebral infarction following angina pectoris might be clearly revealed.

## 2. Case Presentation

**Figure 1:**
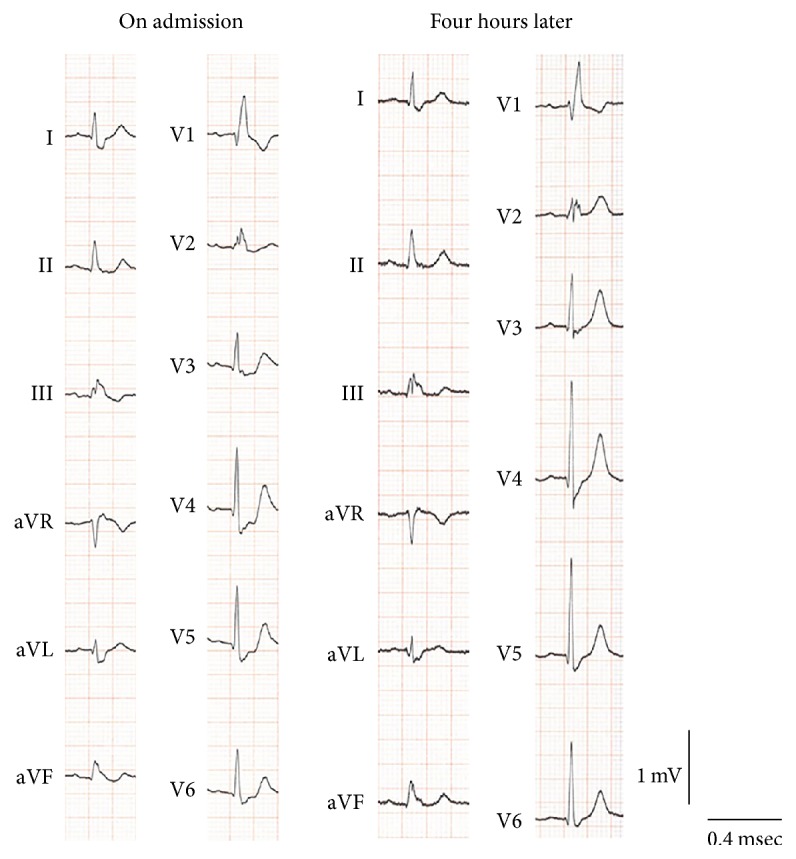
An electrocardiogram (ECG) showed complete right bundle branch block and ST-segment depression in II, III, aVF, and V2 to V6 on admission. ST-segment depression had disappeared when an ECG was redone 4 h later.

**Figure 2:**
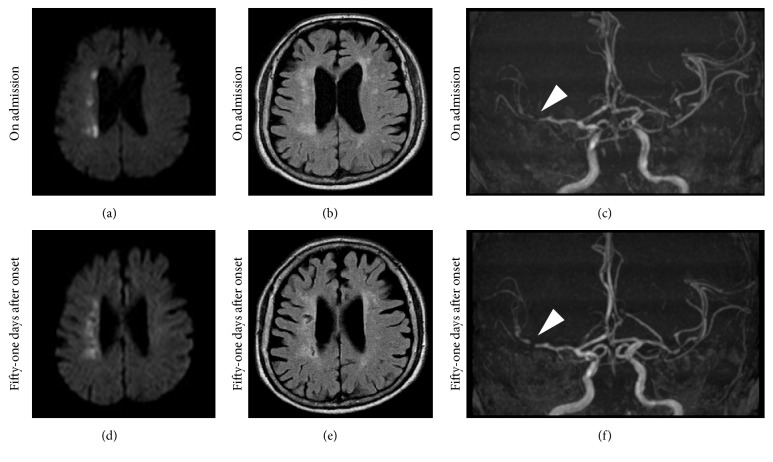
Magnetic resonance imaging (MRI) and angiography (MRA). (a) Diffusion-weighted image (DWI) on admission showed a high intensity lesion in the watershed area of the right anterior and middle cerebral arteries. (b) Fluid attenuated inversion recovery (FLAIR) on admission showed a high intensity lesion in the same area as the DWI. In addition, there were some other high intensity lesions in the deep white matter that reflected old lacunar infarctions. (c) MRA showed the stenosis of the distal portion of the right middle cerebral artery (arrowhead). (d) On the 51st day after onset, a DWI showed that the lesion was still high. (e) FLAIR on the 51st day after onset showed some of the new lesions became very low. (f) MRA on the 51st day after onset showed stenosis of the same portion of the right middle cerebral artery (arrowhead). ((a), (b), and (c)) On admission. ((d), (e), and (f)) 51 days after onset.

**Figure 3:**
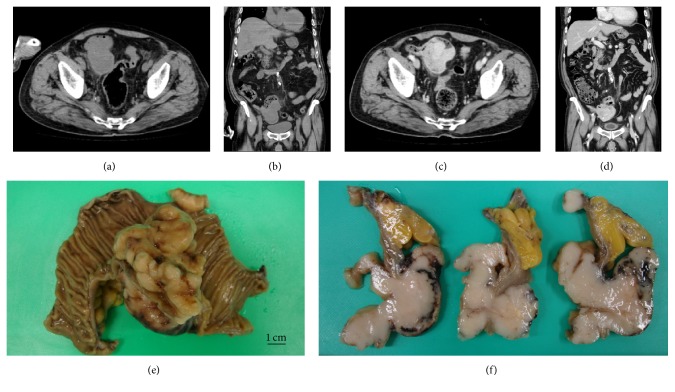
The computed tomography (CT) showed a mass in the small intestine with heterogeneous enhancement. A curative surgery was performed on the 21st day of admission. The tumor had adhered to the retroperitoneum because of localized peritonitis. The tumor size was 60 × 58 × 55 mm. ((a), (b)) Plain CT ((a) axial view, (b) coronal view). ((c), (d)) Enhanced CT ((c) axial view, (d) coronal view). (e) Gross appearance of the resected mass. (f) sections of the mass. Scale bar = 1 cm.

A 75-year-old man with general fatigue 6 months prior to admission showed chest discomfort followed by disturbances in consciousness and was transferred to our ambulance center. He had a past history of hypertension and had been taking antihypertensive drug until admission. His only coronary heart risk factor was hypertension. On admission, he showed consciousness disturbance (E1V3M5: Glasgow coma scale) and left hemiplegia. His blood pressure was 103/64 mmHg, heart rate 110 beats/min, and body temperature 39.0°C. His blood tests showed a white blood cell count of 12.7*∗*10^3^/*μ*L, a hemoglobin level of 5.0 g/dL, a hematocrit level of 17.6%, a platelet level of 343*∗*10^3^/*μ*L, troponin T positivity, a creatine kinase level of 60 U/L, a low-density lipoprotein cholesterol level of 98 mg/dL, a triglyceride level of 66 mg/dL, and a C-reactive protein (CRP) level of 7.87 mg/dL. The electrocardiogram (ECG) on admission showed complete right bundle branch block and ST-segment depression in II, III, aVF, and V2 to V6. ST-segment depression had normalized in ECG redone 4 hours later (Figure 1). The ultrasound cardiogram (UCG) showed an ejection fraction of 60% and mild hypokinesis in the apical portion of the left ventricle which had also normalized in a UCG repeated on the 8th day of admission. Magnetic resonance imaging showed cerebral infarction of the watershed area between right anterior and middle cerebral arteries. A magnetic resonance angiography showed stenosis of the distal portion of right middle cerebral artery (Figure 2). Computed tomography (CT) showed a mass in the small intestine with heterogeneous enhancement, surrounded by mesenteric fat with hyperdensity indicating localized peritonitis (Figures 3(a)–3(d)).

On the next day of admission, the patient regained full consciousness. Creatine kinase showed 90 U/L on the 2nd day and 23 U/L on the 6th day of admission. He was transfused with 4 units (equivalent to 800 mL of total blood) of red blood cell concentration to treat the severe anemia and the hemoglobin levels and hematocrit were 8.2 g/dL and 28.1%, respectively, on the 6th day of admission. After the transfusion, the patient was given an iron preparation until the surgery on the 21st day of admission. The hemoglobin levels and hematocrit increased to 9.9 g/dL and 32.0%, respectively, on the 20th day of admission. The cerebral infarction was treated using glycerol and edaravone without antiplatelets without antiplatelet therapy because of the risk of gastrointestinal hemorrhage. The patient was also treated using antibiotics because of the high fever and hyper-CRPemia, which might have been caused by the localized peritonitis around the intestinal tumor. Body temperature was normalized on the 3rd day of admission and CRP levels reduced to 0.67 mg/dL on the 8th day of admission.

**Figure 4:**
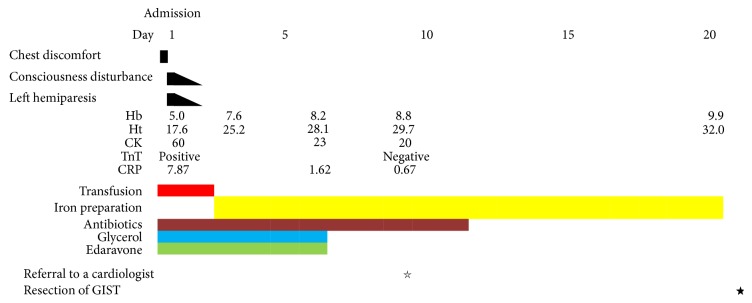
Clinical course.

After his functional capacity was evaluated by a cardiologist, the patient demonstrated more than 4 METs on a treadmill test. On the 21st day of admission, a curative resection was performed. The tumor was located 25 cm from terminal ileum on the oral side, and it penetrated to the retroperitoneum and caused localized peritonitis. Part of the ileum had adhered to the retroperitoneum because of the inflammation, though this could be detached manually. The tumor size was 60 × 58 × 55 mm (Figures 3(e) and 3(f)). A pathological analysis showed that the tumor was composed of spindle cells with fascicles and whorls which is typical for GISTs. The left hemiplegia gradually disappeared and the patient was discharged from the hospital without complications on the 35th day of admission. The patient was going to be readmitted to a heart center and undergo coronary angiography to evaluate the risk for angina pectoris. The clinical course of the patient is summarized in a scheme (Figure 4).

## 3. Discussion

**Figure 5:**
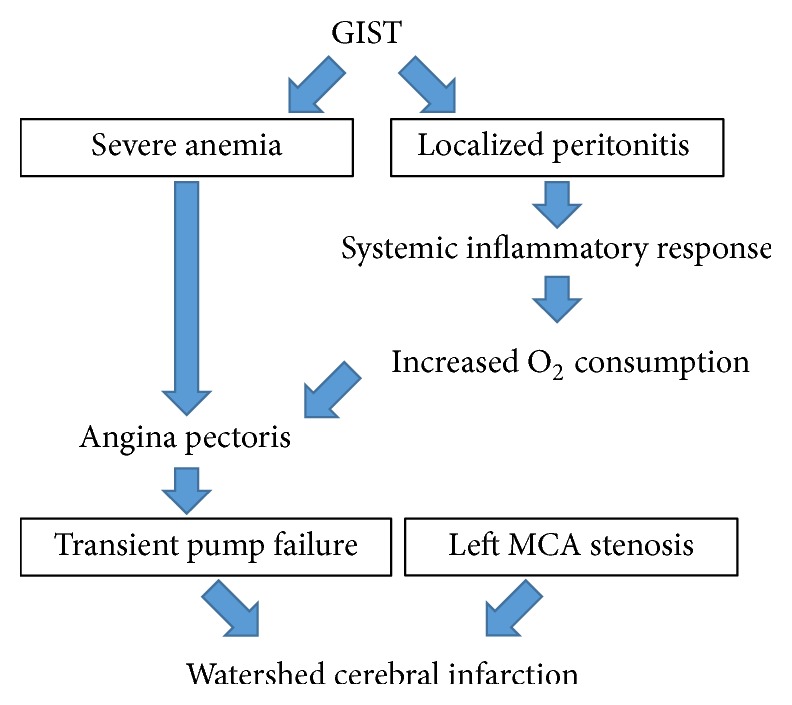
Scheme of the possible pathophysiology in this case.

On admission, it was difficult to diagnose the pathophysiology of this patient. In this patient, the medical history showed consciousness disturbance and left hemiplegia following chest discomfort, which meant that angina pectoris occurred prior to cerebral infarction. We also found the transient ST-segment depression in II, III, aVF, and V2 to V6 through an ECG on admission that disappeared within a few hours. Anemia is a risk factor for ischemic heart disease and a lower level of hemoglobin is associated with its severity [6]. Fever and severe anemia might increase oxygen consumption of the myocardium and provoke the development of angina pectoris (Figure 5). In general, ST-segment elevation provides information on where a lesion is located, and ST-segment depression recorded by the precordial lead does not specify the location of a lesion [7]. In this case, because the apical portion of the left ventricle showed mild hypokinesis, the distal portion of the anterior descending branch might be the culprit artery. The patient was required to undergo a coronary angiography to specify culprit artery for angina pectoris; however, coronary stent implantation required the patient to take dual antiplatelet therapy to prevent stent thrombosis [8]. In addition, the patient demonstrated no cardiac symptoms after admission and more than 4 METs on a treadmill test, so we hypothesized that the main cause of the angina pectoris was the severe anemia and the localized peritonitis. Generally, it is not useful to perform further cardiac imaging for patients with moderate to good functional capacity (≥4 METs) in the perioperative period [9]. Then, we decided to perform curative resection of the GIST that had caused intestinal hemorrhage first. Antiplatelet therapy was initiated soon after the surgery was successfully performed. The balance between antithrombotic therapy and treatment of gastrointestinal bleeding is challenging. In one case, a patient with myocardial infarction began dual antiplatelet therapy and showed active bleeding from colon cancer. Though a stent was placed in his right coronary artery, it was occluded after the dual antiplatelet therapy was discontinued for the abdominal surgery [10]. His partial colonic resection was successfully performed. However, it is often hard to construct a treatment strategy for such complex cases.

Although GISTs are a rare type of cancer, they are the most common sarcoma in the gastrointestinal tract. Surgery remains the only modality that can offer a permanent cure of GISTs [3]. Trousseau's syndrome is a well-known cancer-associated thrombosis that was first described in 1865 by Armand Trousseau [11]. However, a direct link between GISTs and thrombosis has not been previously reported.

In this case, the stenosis of the distal portion of the right middle cerebral artery would be the culprit lesion of the watershed cerebral infarction. At present, clinical studies have not shown that the stent implantation for intracranial arterial stenosis is beneficial for the secondary prevention of ischemic stroke. Recently, in the Stenting Versus Aggressive Medical Therapy for Intracranial Arterial Stenosis (SAMMPRIS) trial, 451 patients with recent transient ischemic attack or stroke related to 70–99% stenosis of a major intracranial artery were randomly assigned to aggressive medical management or aggressive medical management plus stenting using the Wingspan stent. As a result, during a median follow-up of 32 · 4 months, 34 (15%) of 227 patients in the medical management group and 52 (23%) of 224 patients in the stenting group had died or had a stroke [12]. This study tells us that patients should be carefully selected for intracranial artery stenosis stenting until an improved device for stenting exists.

Overall, the severe anemia (hemoglobin 5.0 g/dL) and localized peritonitis of the patient in the current report could have been the main trigger for his angina pectoris because he had no cardiac symptoms, demonstrated more than 4 METs on a treadmill after transfusion, and underwent curative surgery for a GIST without complications. However, the patient suffered hypertension, which is a coronary risk factor, and so an assessment of coronary stenosis was appropriate.
